# Lumbar spinal epidural lipomatosis: A case report and review of the literature

**DOI:** 10.1016/j.ijscr.2020.11.128

**Published:** 2020-11-30

**Authors:** Fabrice Mallard, Manar Buni, Paul S. Nolet, Peter Emary, John A. Taylor, Gemah Moammer

**Affiliations:** a Division of Graduate Education and Research, Canadian Memorial Chiropractic College (CMCC), Toronto, Ontario, Canada; b Institut Franco-Européen de Chiropraxie, Toulouse, France; c Faculty of Medicine, University of Szeged, Szeged, Hungary; d Care and Public Health Research Institute, Maastricht University, Maastricht, Netherlands; e Department of Health Research Methods, Evidence and Impact, McMaster University, Hamilton, Ontario, Canada; f Private Practice, Cambridge, Ontario, Canada; g D'Youville College, Department of Chiropractic, Buffalo, New York, USA; h McMaster University, Grand River Hospital, Kitchener, Ontario, Canada; i Spine Surgery, Grand River Hospital Corporation, Kitchener Waterloo Site, Ontario, Canada; j Spine Surgery, St Mary's General Hospital, Kitchener, Ontario, Canada

**Keywords:** Case report, Spinal epidural lipomatosis, Low back pain, Posterior spinal decompression, Posterior spinal instrumented fusion, Conservative care

## Abstract

**Introduction:**

Lumbar spinal epidural lipomatosis (SEL) is a rare condition defined by an excessive deposition of adipose tissue in the lumbar spinal canal. The objective of this case report is to document a clinical case of SEL presenting within a multidisciplinary spine clinic and to compare our clinical findings and management with the available literature.

**Case presentation:**

A 51-year-old female presented at a spine clinic with low back pain, bilateral leg pain and difficulty walking. Magnetic resonance imaging of the lumbar spine showed evidence of severe central canal stenosis due to extensive epidural lipomatosis. She was initially advised to lose weight and undergo a 3-month course of physiotherapy. However, because of lack of improvement, she was scheduled for and underwent L4-S1 posterior spinal decompression and L4-L5 posterior spinal instrumented fusion. At 12-month follow-up, the patient reported no pain and retained the ability to walk regular distances without experiencing discomfort.

**Discussion:**

This case report describes the conservative and surgical management of a case of lumbar spinal stenosis due to SEL. The therapeutic approach of patients with this condition is not standardized. As such, a discussion of the literature with respect to the diagnosis, clinical presentation, epidemiology, imaging appearance, risk factors, etiology, and management of SEL is also presented.

## Highlights

• Lumbar spinal epidural lipomatosis (SEL) is a rare cause of low back and lower extremity pain.
• Magnetic resonance imaging is considered as the reference standard for the diagnosis.
• The therapeutic approach of patients with SEL can be either surgical or conservative depending on the etiology and the severity of the condition.

## 1 Introduction

Lumbar spinal epidural lipomatosis (SEL) is a rare condition defined by an excessive deposition of adipose tissue in the lumbar spinal canal. Patients will present with low back pain and possible neurogenic claudication with bilateral or single leg pain, paresthesia and/or weakness. The diagnosis is confirmed by magnetic resonance imaging (MRI) [1]. Patients are commonly misdiagnosed because of the atypical clinical presentation and treated for lumbar degenerative spinal stenosis.

The first line of recommended treatment is usually conservative care, but surgical interventions will be suggested to patients in cases of severe or progressing neurological findings and/or absence of improvement of clinical symptoms with conservative care [2].

The objective of this case report is to document a clinical case of SEL presenting within a multidisciplinary spine clinic. This work has been reported in accordance with the Surgical CAse REport (SCARE) guidelines [3].

## 2 Presentation of case

A 51-year-old female presented at a multidisciplinary spine clinic with low back pain and bilateral leg pain. Her pain progressed gradually over many years and was mechanical in nature; standing upright and walking for 100 m (5 min) aggravated her symptoms while sitting and bending forward relieved her symptoms. The patient denied any signs of cauda equina syndrome.

On general observation the patient appeared to be overweight and in mild distress due to back pain. During physical examination, the patient could not walk on her toes or heels. On palpation, her extremities exhibited normal vascular pulses. She also displayed normal and nontender spinal range of motion. When performed in the seated position, her neurological exam, including motor strength, sensory examination, deep tendon reflex, and straight leg raise was normal.

**Fig. 1:**
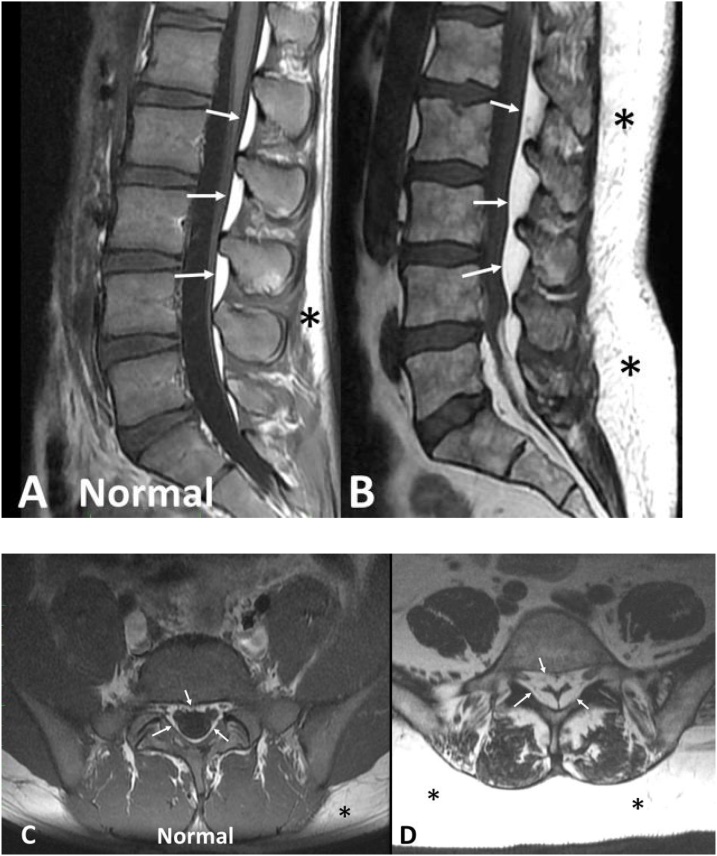
A,B: Sagittal T1-weighted MR images. A. Normal patient image for comparison; B. Patient image. The image of our patient (B) reveals a thick layer of abundant subcutaneous fat (*) and a thick proliferation of epidural fat (arrows) compressing and displacing the dural sac toward the anterior. Observe on the normal image (A) that both subcutaneous fat and epidural fat are less abundant and cause no compression or displacement of the dural sac. C,D: Axial T1-weighted MR images. C. Normal patient image for comparison; D. Patient image. Image C of a normal patient shows a very thin rim of epidural fat surrounding the circular shaped dural sac with no evidence of compression. The axial image of our patient (B) shows dramatic proliferation of epidural fat (arrows) compressing the dural sac into a trefoil shape to approximately 25% of its normal size. Note also the abundance of subcutatneous fat (*) in our patient compared to the normal patient.

MR imaging of the lumbar spine showed L4-L5 and L5-S1 degenerative disk disease with evidence of severe central canal stenosis due to extensive epidural lipomatosis (Fig. 1).

The patient was scheduled for follow-up with the surgeon within 3 months of the initial consultation. Within this period, she was advised to lose weight, undergo a course of physiotherapy, and consult with a physiatrist for pain management services (lidocaine injections).

**Fig. 2:**
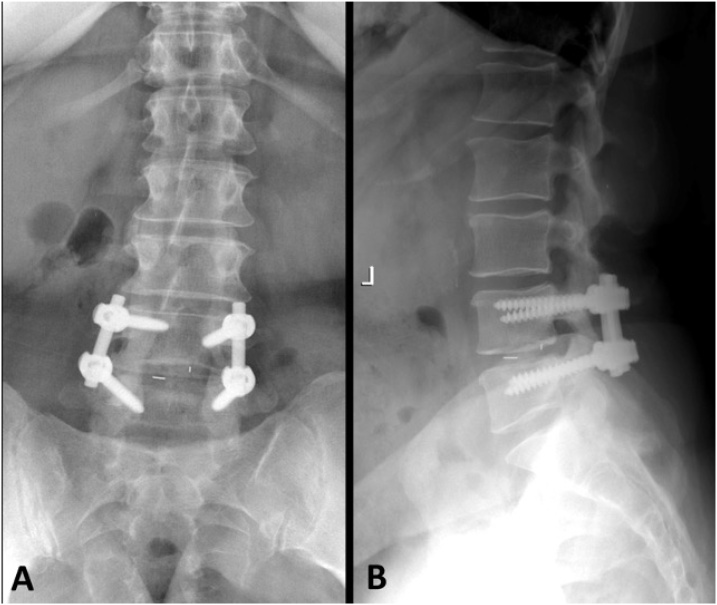
Postoperative frontal (A) and lateral (B) lumbosacral radiographs reveal absence of spinous processes and laminae at L4 and L5 representing evidence of laminectomy. Bilateral pedicle screw and rod instrumentation has resulted in L4-5 arthrodesis (fusion) and small surgical clips are present within the L4-5 disc space related to discectomy at this level.

By 3 months, the patient’s symptoms had not improved with the suggested non-operative treatment. Her left hip radiographs showed severe osteoarthritis (OA) in the hip joint and the patient was subsequently referred to an orthopedic surgeon for consultation. Flexion-extension radiographs of the lumbar spine revealed facet joint osteoarthrosis and intersegmental instability at L4-L5. Based on the patient’s diagnostic imaging and clinical findings, she was scheduled for L4-S1 posterior spinal decompression and L4-L5 posterior spinal instrumented fusion (Fig. 2). This procedure was performed by a senior spinal surgeon. No medication was used for epidural lipomatosis; however pain killers were given for the lower back and leg pain symptoms. Two weeks after surgery, the patient reported that her back and leg pain severity had reduced to 4/10. She was then seen for follow-up at 6 weeks, 3 months and 1 year in the hospital. During the follow-up visits, the level of leg and low back pain through a numerical pain rating scale and the walking abilities were used as outcome measures. At last follow-up, she reported no pain and retained the ability to walk regular distances without experiencing discomfort.

## 3 Discussion

### 3.1 Clinical presentation

Back pain is among the most frequently reported symptoms by patients with SEL [4]. Complaints are usually slowly progressive in nature. Patients can present with typical signs of claudication, motor weakness, radiculopathy, or myelopathy. Some patients may also present with signs of cauda equina syndrome and/or paraplegia that would require urgent medical treatment. This has been reported but it is a rare complication [4].

### 3.2 Etiology and risk factors

Fogel et al. identified five main categories to explain the etiology of SEL: exogenous steroid use (55.3% of cases), obesity (24.5%), surgery induced, idiopathic, and endogenous steroid hormonal disease [2]. SEL has been reported in individuals who have had ongoing steroid therapy, local corticosteroid injections, Cushing’s syndrome, obesity, carcinoid tumor and hypothyroidism [[4], [5], [6], [7]]. Exogenous steroid use is consistently reported as the most significant risk factor for developing SEL. In a cross-sectional study, risk factors significantly associated with the presence of SEL were male sex (OR: 2.01, 95% CI: 1.71–2.37), body mass index (BMI) >30 (OR: 2.59, 95% CI: 1.97–3.41), Black/African American race (OR: 1.66, 95% CI: 1.24–2.23), systemic corticosteroid use (OR: 2.59, 95% CI: 1.69–3.99), and epidural corticosteroid injections (OR: 3.48, 95% CI: 2.82–4.30) [8]. In a case-control study of 199 subjects with SEL and 199 matched controls from a large academic tertiary care referral center [9], risk factors for SEL were history of smoking (OR: 1.90, 95% CI: 1.23–2.94), diabetes mellitus type 2 (OR: 2.17, 95% CI: 1.33–3.56) or being on disability (OR: 4.43, 95% CI 2.48–7.91). Some other risk factors have also been reported. A case of SEL associated with highly active antiretroviral therapy in an HIV patient has been reported in the literature [10]. Some authors also suggested a correlation between Scheuermann’s disease and SEL [11]. There are also suggestions that use of androgen antagonist agent may be associated with the development of SEL in patients with prostate cancer [12].

### 3.3 Epidemiology

SEL was first described in 1975 [13]. SEL is an overgrowth of epidural adipose tissue in the spinal canal and has been reported to be a rare cause of back and leg pain [14]. Clinical charts of 28,902 individuals with spine MRI reports, 18 years of age and older showed a prevalence of SEL of 2.5% with 0.6% being incidental, 1.8% having associated spinal symptoms and 0.1% having symptoms specific to SEL [15]. In 831 patients diagnosed with spinal stenosis in a community spine surgeon’s clinic [16] the prevalence of moderate to severe symptomatic SEL was 6.3% and the annual incidence was 2.5%. The average age of diagnosis of SEL was 62 years of age and the most common level in the lumbar spine for SEL was the L5-S1 level [16].

### 3.4 Differential diagnosis

Because patients most commonly present with low back pain and signs of neurogenic claudication, SEL must be differentiated from spinal stenosis due to degenerative changes. Other conditions that would lead to neurological compression (e.g. spinal fracture, spinal angiolipoma, abscess, spinal tumors, disc bulge, ligamentum flavum hypertrophy, paraspinal lesions, or epidural metastases) could also present with the same clinical presentation [17].

### 3.5 Histology

The pathophysiology has not been well described in the literature [18]. In SEL, tissue will present as epidural unencapsulated hypertrophic fat that will directly compress the neural structures [1,19].

### 3.6 Imaging appearance

MRI has been shown to be the most sensitive imaging modality to observe and evaluate SEL [1]. MRI is useful in differentiating SEL from degenerative changes in patients presenting with clinical signs of spinal stenosis.

The Borré method [1] was used to grade the SEL on MRI in this case report. It is a four grade (0, 1, 2 and 3) scale based on the ratio between the measurements of the dural sac, epidural fat and antero-posterior diameter of the spinal canal. The SEL was graded for the current patient as grade 3 (see Table 1). In terms of correlating imaging findings of SEL to clinical symptoms, in a 2013 study by Patel et al. [5] all patients with Borré grade 1 SEL on MRI were clinically asymptomatic, whereas all patients with grade 3 SEL were symptomatic with clinical signs of severe spinal stenosis. By comparison, 17% of patients with grade 2 SEL were also reported as symptomatic [5]. The intraobserver and interobserver agreement for reliability of this classification method have shown a kappa coefficient ranging from 0.79 (95% CI, 0.65–0.93) to 0.82 (95% CI, 0.70–0.95) and from 0.76 (95% CI, 0.62–0.91) to 0.85 (95% CI, 0.73–0.97) respectively [1].

**Table 1:** MRI grading of lumbar spinal epidural lipomatosis from Borré et al. [1].

Grade	DuS/EF index	EF/SpiC index (%)	Observation
Normal 0	≥1.5	40	Normal amount of epidural fat
Grade 1	1.49–1	41–50	Mild overgrowth of epidural fat
Grade 2	0.99–0.34	51–74	Moderate overgrowth of epidural fat
Grade 3	≤0.33	75	Severe overgrowth of epidural fat

DuS = dural sac; ES = epidural fat; SpiC = spinal canal.

Recently, a new method has been developed to assess the total epidural fat volumes throughout the lumbar spine [20], termed by Walker et al. as the “Fat Finder” algorithm. The preliminary evidence, on a sample of 11 patients, showed an intraclass correlation coefficient (ICC) that ranged from 0.993 to 0.999 for intraobserver reliability and from 0.997 to 0.998 for interobserver reliability. However, more studies are needed to confirm the validity and reliability of this method.

### 3.7 Treatment

The treatment of SEL will depend upon the etiology and the severity of clinical signs. If the etiology is known, the treatment will be adapted to target the condition [21].

Surgical intervention will be indicated in cases of failure of conservative treatment or in the presence of progressive and severe neurological deficits. This will include a decompressive laminectomy and resection of epidural adipose tissue [2,22]. Spinal canal decompression and lipomatosis excision could also be another surgical option. In the current case, the patient underwent posterior spinal decompression and instrumented fusion with favorable results.

In a literature review, Fogel et al. [2] estimated a success rate of 77% with surgical intervention of patients with SEL due to steroid use. In a similar population of patients, Borré et al. [1] reported a success rate of 75% in cases when treated with conservative care or surgical intervention. In the Borré et al. study, the authors did not provide conclusions on specific criteria to aid clinicians in selecting an appropriate operative or non-operative treatment modality. Our patient had been referred to a pain clinic for physiotherapy services before the surgical treatment. However, because she did not improve sufficiently she was referred for surgical intervention.

In a population of 104 patients with SEL [2], the success rate of surgery in obese patients was 66.7% and the success rate of conservative management in obese patients with a focus on weight loss was 81.8%. Based on these findings, Fogel et al. suggest that weight loss may be considered as a first line of treatment for SEL in this population of patients [2]. Considering the evidence in the current case, the medical team suggested to the patient to lose weight. This may have had positive consequences on her recovery but was not sufficient to avoid surgical intervention. Furthermore, there is a current lack of evidence regarding the combination of conservative and operative care to improve the clinical symptoms of patients with SEL.

Following surgical intervention, the prognosis of idiopathic SEL has been reported as favorable. For example, Ferlic et al. [23] reported only one case of recurrence of symptoms and imaging findings in a series of 22 surgically treated SEL patients at long-term (i.e. 7-year) follow up. The surgical intervention of our patient was successful with an increase in walking distance and a decrease of pain. Longer term follow-up of our patient will be needed however to see if the patient’s outcomes are stable and to evaluate the risk or presence of recurrence. To date, much of the literature on SEL for surgically and non-surgically treated patients has been limited to case reports and case series [2]. As such, future observational studies evaluating long-term outcomes and comparing interventions for SEL management are needed.

Currently, there are also no valid and reliable outcome measures that could be used to assess the success of surgical decompression in this case. Our patient’s improvement was mainly based on the improvement in their walking ability which has been shown to be a valid outcome measure in patients with chronic signs and symptoms associated with lumbar spinal stenosis [24].

Patel et al. [4] reported a spontaneous resolution of SEL symptoms in a 53-year-old man with obesity and history of chronic back pain. However, no other similar case reports have been reported in the literature.

## 4 Conclusion

SEL is a rare cause of back pain with neurological symptoms. It is characterized by the accumulation of excess adipose tissue in the spinal canal, causing symptoms associated with neurologic compression and claudication. MRI is considered as the reference standard for the diagnosis of SEL. The therapeutic approach of patients with SEL is individualized and can be either surgical or conservative depending on the etiology of the condition. Surgical interventions could be reserved for patients with severe symptoms or those who fail conservative management. However, there are currently no published clinical practice guidelines to inform clinicians on the conservative or surgical care for this condition.

## Declaration of Competing Interest

No conflicts of interest.

## Funding

No source of funding.

## Ethical approval

This case report was approved by the Ethics Committee of the Canadian Memorial Chiropractic College. (REB Approval #: 2006X02).

## Consent

Informed consent was obtained from the patient for publication of this case report and accompanying images.

## Author’s contribution

MB: Manar Buni, PN: Paul Nolet, PE: Peter Emary, GM: Gemah Moammer, FM: Fabrice Mallard, JAT: John A. Taylor

Study concept or design – MB, PN, PE, GM.

Data collection – MB, GM.

Data interpretation – MB, PN, PE, JAT, GM.

Literature review – FM, PN, PE, GM.

Drafting of the paper – FM, MB, PN, PE, JAT, GM.

Editing of the paper – FM, MB, PN, PE, JAT, GM.

## Registration of research studies

Clinicaltrials.gov, NCT04622501: https://clinicaltrials.gov/ct2/show/NCT04622501.

## Guarantor

Gemah Moammer.

Fabrice Mallard.

## Provenance and peer review

Not commissioned, externally peer-reviewed.
